# Unbiased Transcriptional Comparisons of Generalist and Specialist Herbivores Feeding on Progressively Defenseless *Nicotiana attenuata* Plants

**DOI:** 10.1371/journal.pone.0008735

**Published:** 2010-01-15

**Authors:** Geetha Govind, Omprakash Mittapalli, Thasso Griebel, Silke Allmann, Sebastian Böcker, Ian Thomas Baldwin

**Affiliations:** 1 Max Planck Institute for Chemical Ecology, Jena, Germany; 2 Faculty of Mathematics and Computer Science, Friedrich Schiller University of Jena, Jena, Germany; University of Liverpool, United Kingdom

## Abstract

**Background:**

Herbivore feeding elicits dramatic increases in defenses, most of which require jasmonate (JA) signaling, and against which specialist herbivores are thought to be better adapted than generalist herbivores. Unbiased transcriptional analyses of how neonate larvae cope with these induced plant defenses are lacking.

**Methodology/Principal Findings:**

We created cDNA microarrays for *Manduca sexta* and *Heliothis virescens* separately, by spotting normalized midgut-specific cDNA libraries created from larvae that fed for 24 hours on MeJA-elicited wild-type (WT) *Nicotiana attenuata* plants. These microarrays were hybridized with labeled probes from neonates that fed for 24 hours on WT and isogenic plants progressively silenced in JA-mediated defenses (N: nicotine; N/PI: N and trypsin protease inhibitors; JA: all JA-mediated defenses). *H. virescens* neonates regulated 16 times more genes than did *M. sexta* neonates when they fed on plants silenced in JA-mediated defenses, and for both species, the greater the number of defenses silenced in the host plant (JA > N/PI > N), the greater were the number of transcripts regulated in the larvae. *M. sexta* larvae tended to down-regulate while *H. virescens* larvae up- and down-regulated transcripts from the same functional categories of genes. *M. sexta* larvae regulated transcripts in a diet-specific manner, while *H. virescens* larvae regulated a similar suite of transcripts across all diet types.

**Conclusions/Significance:**

The observations are consistent with the expectation that specialists are better adapted than generalist herbivores to the defense responses elicited in their host plants by their feeding. While *M. sexta* larvae appear to be better adapted to *N. attenuata'*s defenses, some of the elicited responses remain effective defenses against both herbivore species. The regulated genes provide novel insights into larval adaptations to *N. attenuata'*s induced defenses, and represent potential targets for plant-mediated RNAi to falsify hypotheses about the process of adaptation.

## Introduction

The co-evolution of plants and insects has primarily been driven by their interactions [1], [2], [3], [4]. Plants respond to herbivore attack with highly evolved, elegantly regulated arrays of responses. Attack triggers at least two types of inducible defense responses: those that involve the production of metabolites that directly retard the growth and development of the herbivores (direct defenses) and those that involve the production of metabolites that indirectly protect plants by attracting the herbivores' natural enemies, usually parasitoids and predators (indirect defenses) [5], [6], [7]. These inducible defense mechanisms are tightly regulated by insect elicitors, likely to curtail the costs of production in the absence of herbivory and to prevent insects from adapting to the plant's defenses.

The elicitors found in the oral secretions and regurgitants (OS) of the caterpillars enable plants to specifically recognize attack from insects; this recognition is mediated by complex signaling pathways in which jasmonic acid (JA) plays a central role [7], [8], [9]. The small quantities of OS that are transferred to leaves by the larvae during feeding are sufficient to elicit defense responses [10]. The salivary components are complex and differ among different insect species and some of these differences allow plants to tailor their defense responses to attack from different insect species [11], [12], [13]. The defense responses elicited in plants by these elicitors are also highly complex, frequently involving the production of metabolites from many different biosynthetic pathways that sometimes function synergistically to confer resistance [14], [15], [16], [17], [18]. The attacking insects, on the other hand, do not remain passive, but up-regulate detoxification systems and employ various behavioral responses to counter the plant's defense responses [18], [19], [20]. These counter defense responses are particularly well studied in specialist herbivores that have adapted to their host plant's defenses.

While generalist herbivores are often deterred by the secondary metabolites produced by their host plants, numerous studies reported that many specialists have evolved effective countermeasures [21], [22], [23]. Specialist herbivores often have well-developed specific enzymatic systems that allow them to metabolize secondary chemicals; for example, the specialist bruchid beetle metabolizes toxic non-protein amino acids and synthesizes its own amino acids [24], [25], and the larvae of the specialist *Heliconius* convert cyanogenic glycosides to thiols, which they use as a source of nitrogen [26]. Larvae of the lepidopteran *Papilio* and *Helicoverpa* genera metabolize furanocoumarins with the help of cytochrome-P450-dependent mono-oxygenases [27], [28], [29], [30] and *Manduca sexta* (*Ms*) larvae have developed a greater tolerance for nicotine that exceeds that of other insect species that do not regularly attack nicotine-containing plants [31], [32], [33]. Some specialists can sequester toxic secondary metabolites for their own defense, such as the larvae of the lepidopteran *Uthesia ornatrix*, which sequesters pyrrolizidine alkaloids to defend themselves against predators [34], [35], [36], [37].

The majority of these examples of plant induced defenses and insect counter responses come from studies that examine the responses of adapted insects to single compounds or classes of compounds. Few have examined the responses of insects in an unbiased fashion to the full complement of defenses that are elicited by herbivore attack. This stands in contrast to the many unbiased transcriptional analyses of plant responses to insect attack [38], [39], [40], [41], [42], [43].

*Nicotiana attenuata*, a wild tobacco plant native to the North American Great Basin Desert is regularly attacked by the larvae of specialist (*Ms*), as well as generalist (*Heliothis virescens*: *Hv*) lepidopteran herbivores [44]. This plant's responses to attack from *Ms* larvae have been extensively studied and is known to activate a well-characterized mitogen-activated protein kinase (MAPK) signaling system as soon as it perceives the elicitors, fatty acid-amino acid conjugates, in the OS of *Ms* larvae. This signaling system subsequently triggers JA-, SA-, and ethylene-mediated defense responses [9], [13], [45], [46] that include the accumulation of anti-feedants and secondary metabolites, such as trypsin protease inhibitors (TPIs), nicotine (N), phenolics, putrescine conjugates, diterpene glycosides, etc. [38], [42], [47], [48], [49], [50]. Microarray analysis with a custom microarray enriched in *Ms*-elicited *N. attenuata* genes revealed that there is a large overlap in the transcriptional response to attack from *Ms* and *Hv* larvae [51] despite differences in the biochemical composition of these insects' OS [11], [52], [53].

The genes up-regulated in *N. attenuata* by *Ms* and *Hv* herbivory include the 13-lipoxygenase, *α*-dioxygenase, hydroperoxide lyase, TPI, threonine deaminase, xyloglucan-endotransglycosylase and a WRKY-type transcription factor; all of these are part of *N. attenuata'*s defense response [46], [48], [54], [55], [56], [57]. However, *N. attenuata* plants also elicit different transcriptional signatures in response to attack from these two herbivores. Although attack from both *Ms* and *Hv* larvae activate JA signaling, *Ms* larvae are more tolerant than *Hv* larvae to the defenses that are activated by JA signaling. It is not clear, however, whether these differences result from differences in the responses elicited in the plants by the two species, and/or differences in how these insects respond to the plant's defenses. One way to disentangle these two possibilities is to transform plants to progressively silence their defense responses and query the transcriptional responses of larvae feeding on these progressively defenseless plants in an unbiased manner.

The extensive literature on the physiological mechanisms that *Ms* larvae employ to detoxify N, such as the ‘multi-drug’ pump [58] and P450 enzymes [59], [60], may explain the differences in the response of *Ms* and *Hv* larvae to *N. attenuata'*s defenses, but most of these mechanisms have not been studied in *Hv*. Moreover, it's not clear if these mechanisms are relevant for *Ms*'s tolerance of dietary N as most studies use late instar larvae reared on artificial diets, as their large size greatly facilitate physiological examinations. In nature, however, it is the neonates that must adapt to the specific-defenses of the plant on which their mother oviposited them on. And this process of adaptation must be rapid, as larvae must tolerate the defenses that are elicited during their first meal. An analysis of the transcriptional responses of neonates that are activated in midgut tissues in response to their first meal are likely to reflect the mechanisms by which larvae adapt to host plant defenses [61].

The goal of this study was to compare the global changes in gene expression elicited by the feeding-elicited defenses in the host plant in two native herbivores of *N. attenuata*: the specialist larvae of *Ms* and generalist larvae of *Hv*. To investigate these transcriptional responses, we created cDNA microarrays for each larval species separately by spotting normalized cDNA libraries created from the midguts of neonates that had fed for 24 h on WT *N. attenuata* plants that had been previously elicited by MeJA treatment three days before larval feeding. These microarrays were then hybridized with labeled probes created from RNA extracted from neonates that had fed for 24 h on *N. attenuata* plants progressively silenced in JA-mediated defenses (N, N/PI, all JA-mediated defenses) and compared with probes from neonates that had fed for the same time on WT plants with their full complement of defenses. From the differences in expression patterns in the larvae that fed on the different diets, we draw inferences about the mechanisms by which the larvae adapt to *N. attenuata'*s induced defenses.

## Results

### Experimental Overview

**Figure 1:**
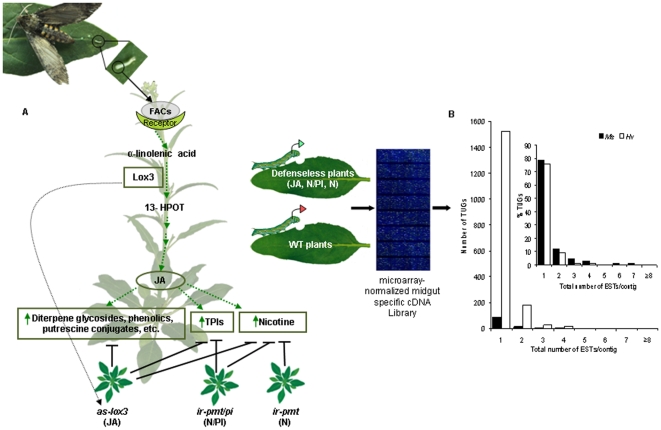
Overview of the strategy used to isolate differentially regulated genes in larvae from a specialist (*Manduca sexta*: *Ms*) and a generalist (*Heliothis virescens*: *Hv*) lepidopteran herbivore of *Nicotiana attenuata*. A) Neonates of *Ms* and *Hv* fed for 24 h on wild-type (WT), untransformed plants and plants transformed to silence: only nicotine (N) biosynthesis, by expressing an inverted-repeat putrescine N-methyl transferase (pmt) construct (*ir-pmt*); both N and trypsin protease inhibitor (N/PI) biosynthesis, by expressing an inverted-repeat pmt and trypsin protease inhibitor (PI) construct (*ir-pmt/pi*); and jasmonic acid (JA) biosynthesis, by antisense-expression of lipoxygenase 3 (*as-lox3*), a specific lox that supplies the JA biosynthetic cascade with fatty acid hydroperoxides. RNA was extracted from the whole larval tissue after neonates fed for 24 h, transcribed into cDNA and labeled with fluorescent dyes (WT-fed larvae: Cy5; N-, N/PI-, JA- fed larvae: Cy3) and hybridized against *Manduca* and *Heliothis* microarrays which had been spotted with midgut specific cDNA library of each species of larvae, normalized using duplex-specific nuclease in the trimmer-direct cDNA normalization kit. Differentially regulated genes (ER: Cy3/Cy5>1.5 or −1/(Cy3/Cy5) <−1.5) were identified, sequenced and analyzed for their putative function. B) The total number of genes regulated by *Hv* larvae (tentative unique genes: TUGs) was 16 times greater than that regulated by *Ms* larvae when both fed on the same defenseless plants, but the percentage of genes in contigs of various sizes (inset) was similar, because both the libraries had been normalized. Graphs show the distribution and percentage of differentially regulated TUGs by *Ms* and *Hv* larvae in contigs of different sizes. Solid bars: *Ms,* open bars: *Hv*.

Both *Ms* and *Hv* larvae introduce some of the same fatty acid-amino acid conjugate elicitors from their OS into plant wounds during feeding and these elicitors induces a specific lipoxygenase (lox3), which catalyzes the oxygenation of α-linolenic acid to 13-hydroperoxides that undergoes further sequential enzymatic reactions to eventually produce JA and its metabolites. These jasmonates, in turn, activate the expression of both direct and indirect defenses. To study the transcriptional changes in *Ms* and *Hv* larvae to JA-mediated defenses elicited and present in their first meal, the larvae of both species were fed on WT plants and defenseless transgenic plants that are progressively silenced in nicotine alone (*ir-pmt*: N); N and TPIs (*ir-pmt/pi*: N/PI); and JA biosynthesis (*as-lox3*: JA), which are impaired in all JA-elicited direct and indirect defenses. Labeled probes prepared from larvae that fed for 24 h on WT and the different, progressively defenseless plants were hybridized to microarrays on which normalized midgut-specific cDNA libraries of *Ms* and *Hv* neonates were spotted separately to identify differentially regulated genes (up-regulated: expression ratio >1.5; down-regulated: expression ratio <−1.5) (Figure 1A).

From these hybridizations, we identified 151 expressed sequence tags (ESTs) which were differentially regulated in *Ms* larvae that could be grouped into 108 tentative unique genes (TUGs). In contrast, *Hv* larvae differentially regulated 2011 ESTs that could be grouped into 1739 TUGs. Hence, when feeding on the same defenseless plants, *Hv* larvae regulated 16 times more transcripts than did *Ms* larvae (Figure 1B). Despite the large difference in the number of transcripts regulated by *Ms* and *Hv* larvae, the percentage of singletons −78.7% and 75.6% from *Ms* and *Hv* larvae, respectively – was almost the same, verifying the normalization of the libraries (Figure 1B, inset).

### Overarching Patterns of Differential Transcript Accumulation

**Figure 2:**
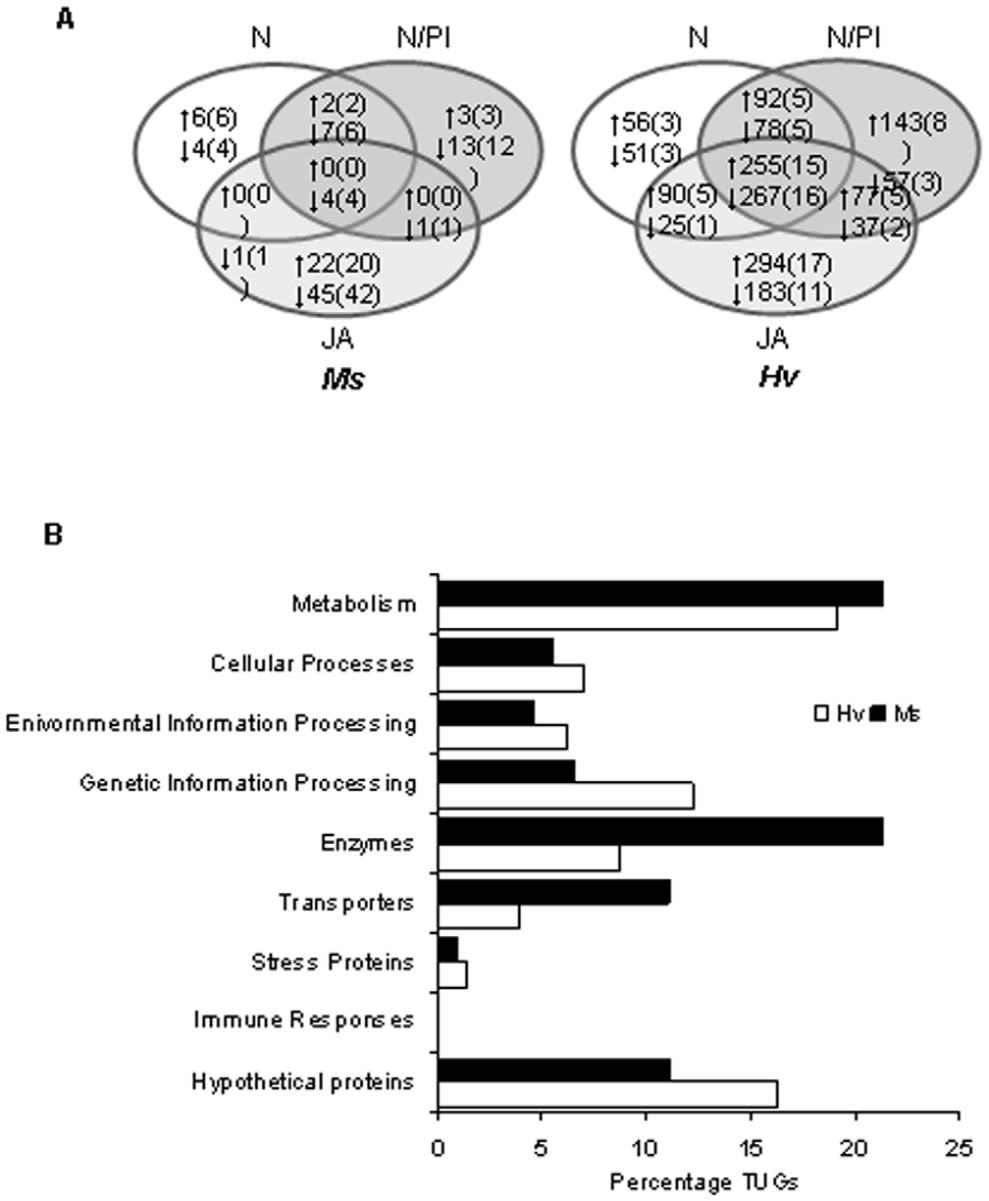
The number of genes regulated by both *Ms* and *Hv* larvae correlated with the number of defense traits that had been silenced in the larval host plants, but more genes were regulated in *Hv* than in *Ms* larvae. A) Venn diagrams depicting the number and percentage of genes significantly up- (ER>1.5) or down-regulated (ER<−1.5) by *Ms* and *Hv* larvae according to which defense compound was silenced in the plants they fed on. When the generalist *Hv* larvae fed on JA-, N/PI-, or N-silenced *N. attenuata* plants 17-, 33- and 38-fold more genes were regulated compared to when the specialist, *Ms* larvae fed on the same plants. Values in parentheses denote the percentage of regulated genes. For both species, the number of genes and the percentage of regulated genes correlated with the number of different direct defenses that had been silenced in the plants: more genes were found to be regulated in larvae that fed on JA-silenced plants than in larvae that fed on N/PI- and N-silenced plants. B) In *Ms* larvae, the percentage of genes coding for enzymes, transporters and metabolism was highly regulated; in *Hv* larvae, the percentage of genes coding for genetic information followed by cellular processes and environmental information processing was highly regulated.

While *Hv* larvae regulated more transcripts than did *Ms* larvae, the overall pattern of regulation in both species tracked the number of defenses that were silenced in the host: the greater the number of defenses silenced in the host plant (JA>N/PI>N), the greater the number of transcripts that were differentially regulated in larvae feeding on these silenced host plants in comparison to larvae that fed on wild-type (WT) plants (Figure 2A). Moreover, the type of regulation differed between the two species: *Ms* larvae down-regulated 2–5 times more transcripts than they up-regulated, but in *Hv* larvae, an equal number of genes were up- and down-regulated. Hence, *Ms* larvae that fed on N-silenced plants regulated 24 transcripts of which 8 were up-regulated and 16 were down-regulated; when they fed on N/PI-silenced plants, they regulated 30 transcripts of which 5 were up-regulated and 25 were down-regulated; and on JA-silenced plants, they regulated 73 transcripts of which 22 were up-regulated and 51 were down-regulated. *Hv* larvae that fed on N-, N/PI- and JA-silenced plants regulated 914 transcripts (493 up-regulated and 421 down-regulated), 1006 transcripts (567 up-regulated and 439 down-regulated) and 1228 transcripts (716 up-regulated and 512 down-regulated), respectively (Figure 2A).

By sequencing these differentially regulated transcripts and mapping them to biochemical pathways, we discovered that *Ms* larvae that fed on defenseless plants regulated more transcripts coding for enzymes (21%), followed by transporters (11%) and metabolism (21%) compared to *Hv* larvae that fed on these defenseless plants (9%, 4% and 19%, respectively). On the other hand, *Hv* larvae regulated more transcripts coding for genetic information processing (12%), followed by cellular processes (7%) and environmental information processing (6%) compared to *Ms* larvae (6%, 6% and 5%, respectively) (Figure 2B).

**Figure 3:**
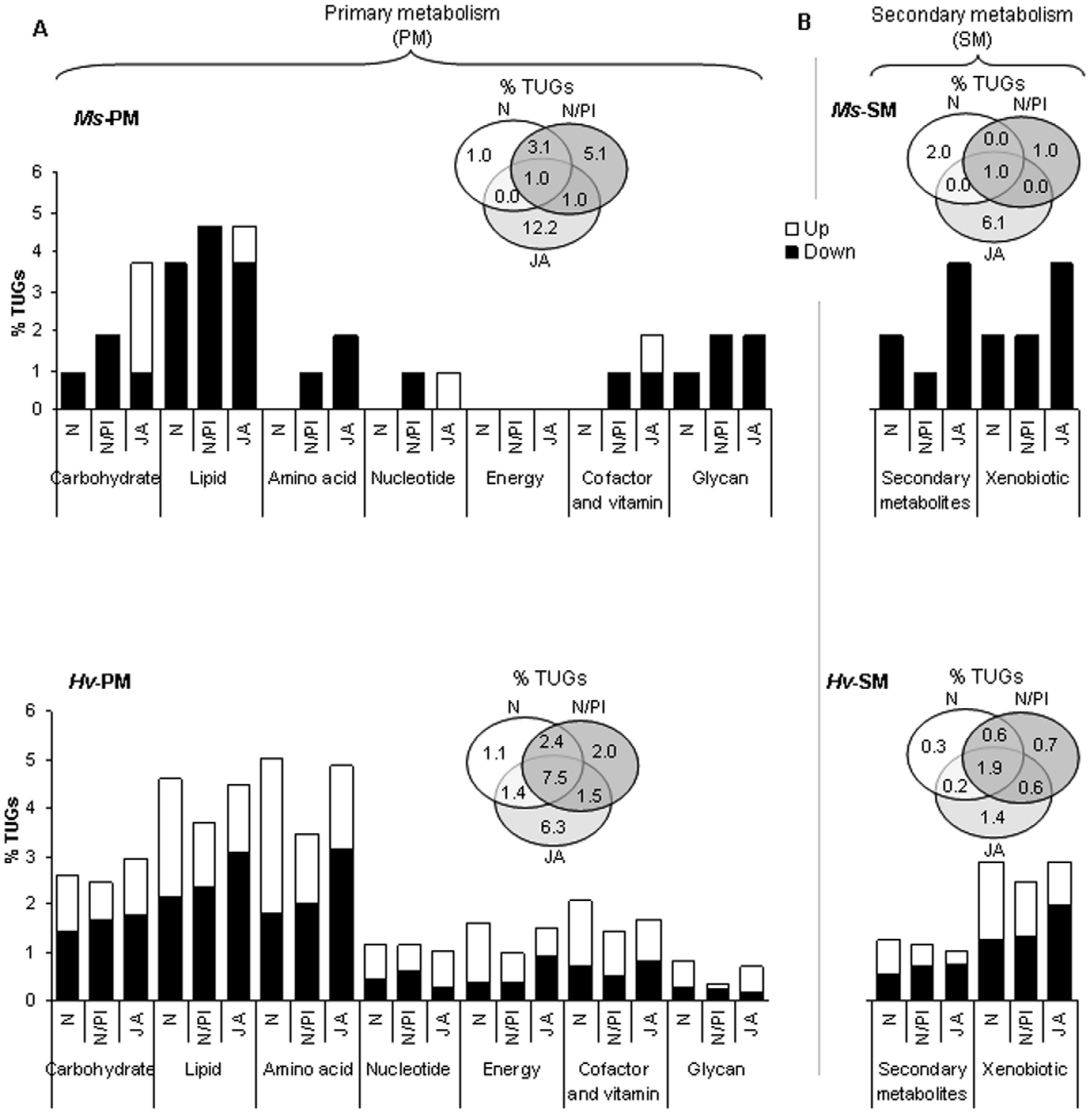
Both *Ms* and *Hv* larvae regulated more genes related to primary and secondary metabolism when they fed on JA-silenced plants than when they fed on N/PI- or N-silenced plants. In *Ms* larvae, these genes were mainly down-regulated while in *Hv* larvae, they were both up- and down-regulated. A) Various functional categories of genes related to primary metabolism were highly regulated in *Hv* larvae, while only a few were regulated in *Ms* larvae; however, *Ms* larvae that fed on JA-silenced plants regulated 1.9-fold more genes than did *Hv* larvae that fed on the same plants. B) In *Ms* larvae, most genes related to secondary metabolism were down-regulated (with percentages down-regulated in larvae fed on JA-silenced plants being the highest followed by N/PI- and N-silenced plants), while in *Hv* larvae, these genes were both up- and down-regulated. *Ms* larvae that fed on JA-silenced plants regulated 4.4-fold more genes than did *Hv* larvae that fed on the same plants. Open bars: up-regulated (ER>1.5); black bars: down-regulated (ER<−1.5). Inset: Venn diagrams depicting the percentage of genes regulated by larvae in response to JA-, N/PI or N-silenced diets; *Ms* (upper panel) and *Hv* (lower panel) for primary (A) and secondary (B) metabolism.

**Figure 4:**
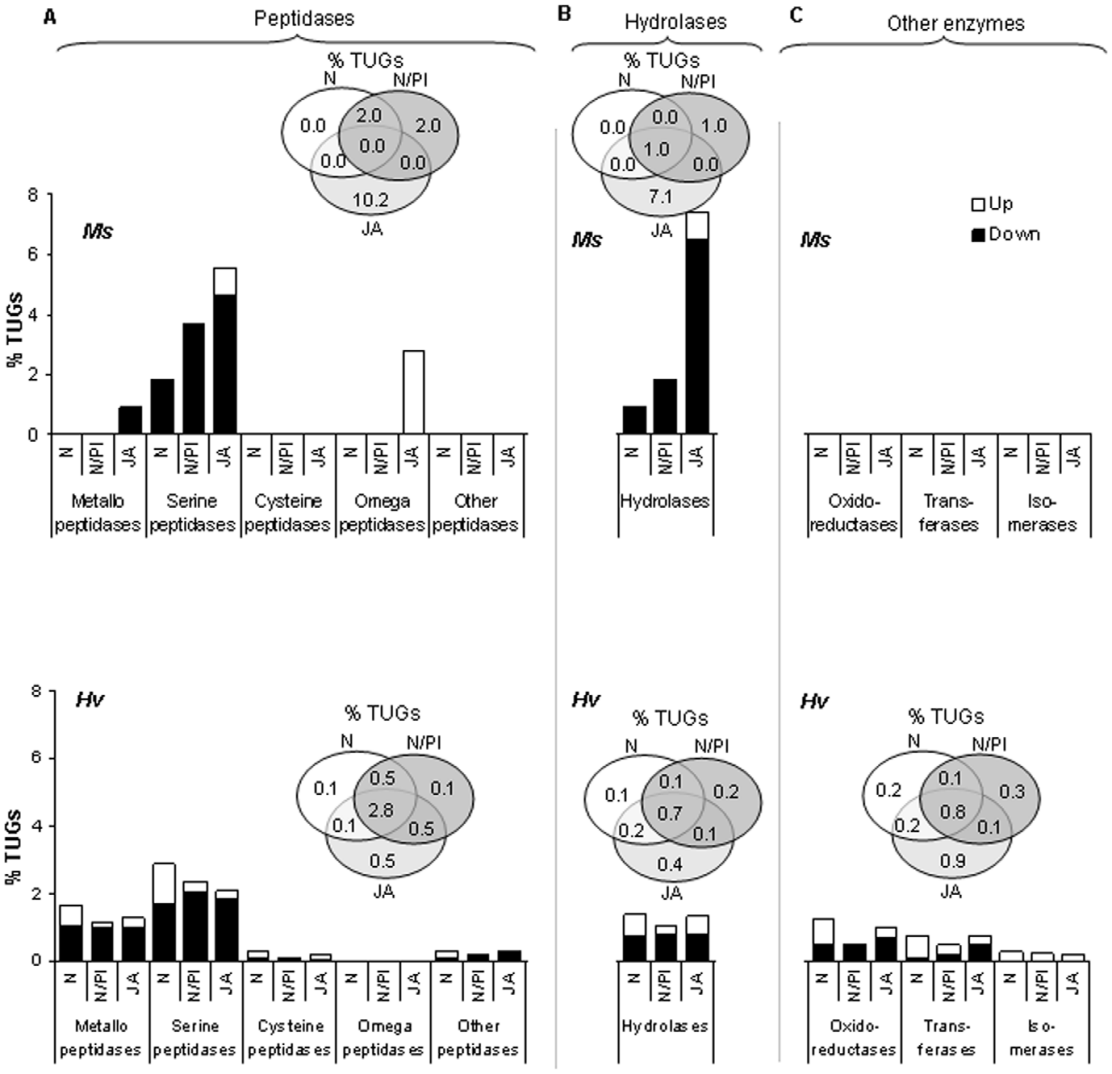
In *Ms* larvae, a higher percentage of peptidases and hydrolases were down-regulated when they fed on plants silenced in JA compared to when they fed on plants silenced in N/PI, and this percentage of down-regulated enzymes was lowest in plants silenced in N; the regulation of these enzyme categories by *Hv* larvae did not follow a pattern organized by defense expression in the host plant. The percentage of genes regulated for A) peptidases was 2.4-fold greater and the B) hydrolases, 5.6-fold greater in *Ms* larvae that fed on plants silenced for JA compared to in *Hv* larvae that fed on the same plants. C) Oxidoreductases, transferases and isomerases (the category referred to as ‘other enzyme’) were regulated only in *Hv* larvae when these fed on JA-, N/PI- or N-silenced plants. Open bars: up-regulated (ER>1.5); solid bars: down-regulated (ER<−1.5). Inset: Venn diagrams depicting the percentage of genes regulated by *Ms* larvae (upper panel) and *Hv* larvae (lower panel) for peptidases (A), hydrolases (B) and other enzymes (C) in response to feeding on variously defenseless plants.

**Figure 5:**
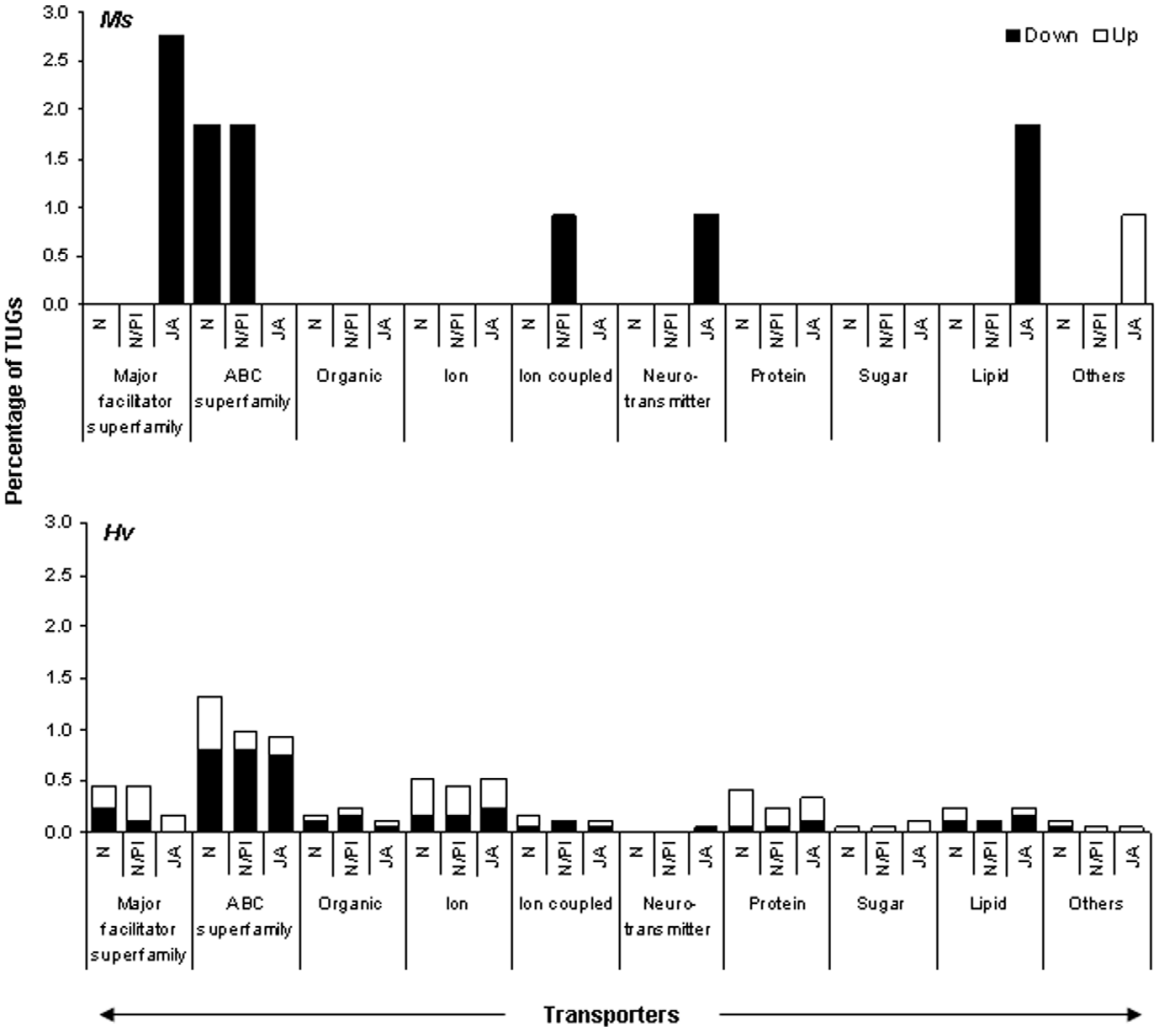
Only a few transporters (mainly MFS and ABC) were down-regulated by *Ms* larvae when the larvae fed on plants silenced for JA-, N/PI- or N; *Hv* larvae up- and down-regulated many transporters. Overall, many transporter genes were down-regulated in *Ms* larvae that fed on JA-silenced plants, fewer were regulated in larvae that fed on N/PI- and N-silenced plants. Major facilitator superfamily transporters and lipid transporters were down-regulated in *Ms* larvae that fed on JA-silenced plants. *Ms* larvae that fed on N/PI- and N-silenced plants down-regulated their ATP-binding cassette (ABC) superfamily of transporter and ion-coupled transporters (upper panel). Few transporter genes were regulated in *Hv* larvae that fed on JA-, N/PI- or N-silenced plants (lower panel). Open bars: up-regulated (ER>1.5); solid bars: down-regulated (ER<−1.5).

**Figure 6:**
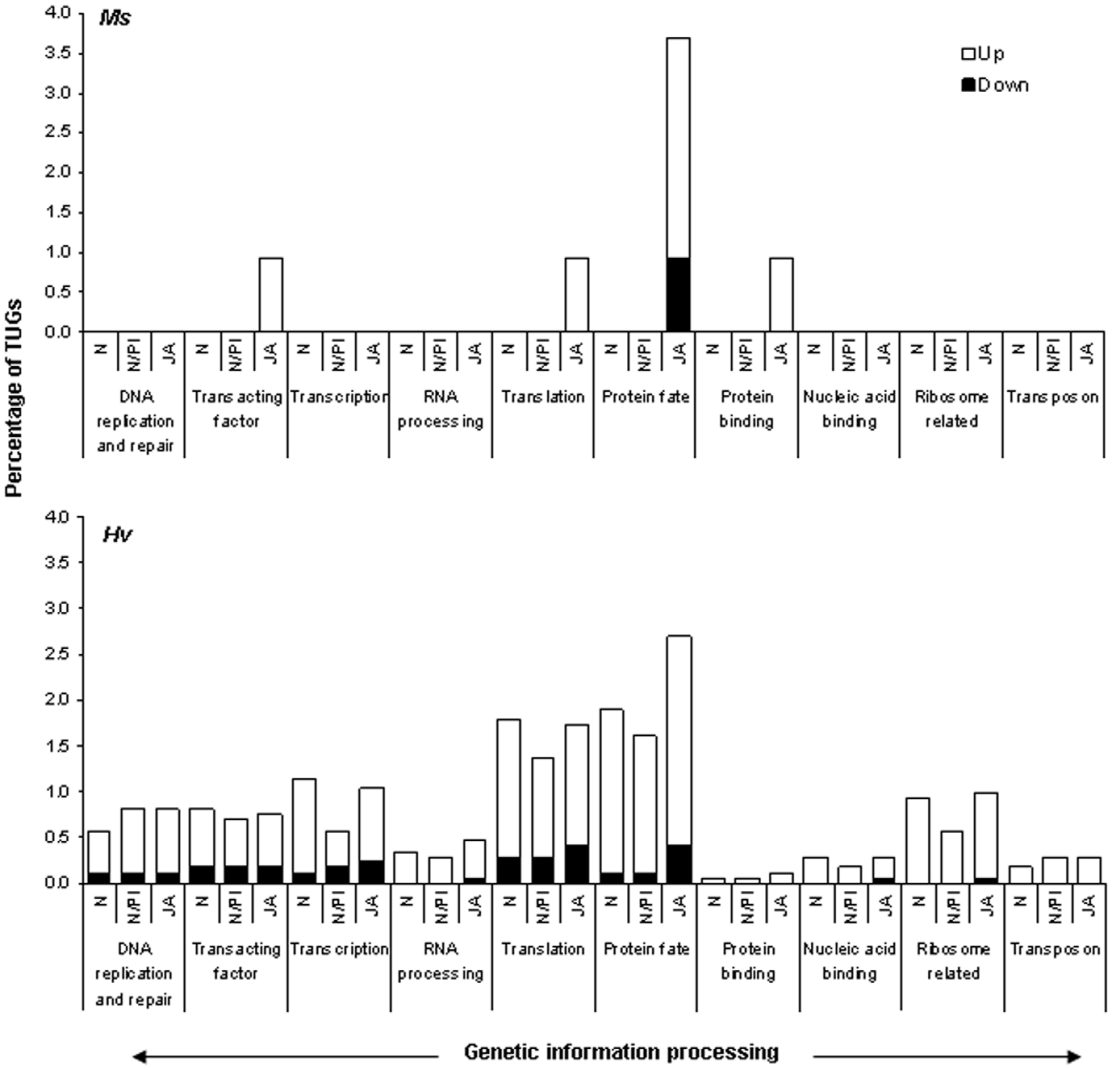
In *Ms* larvae genes for genetic information processing were regulated only when larvae fed on JA-silenced plants; *Hv* larvae regulated genes for genetic information processing when they fed on JA-, N/PI- and N-silenced plants. Open bars: up-regulated (ER>1.5); solid bars: down-regulated (ER<−1.5) by *Ms* (upper panel) and *Hv* (lower panel).

In the following sections, we discuss the differences in the regulation of transcripts in the functional categories of primary and secondary metabolism, peptidase and hydrolases, transporters, and genetic information processing observed in *Ms* and *Hv* larvae that fed on defenseless plants. Two general patterns of regulation were observed. First, while *Ms* larvae down-regulated, *Hv* larvae up- and down-regulated transcripts from the same functional categories. Second, while *Ms* larvae regulated transcripts in a diet-specific manner, *Hv* larvae regulated a more similar suite of transcripts across all diet types (Figures 3, 4, 5, 6, upper and lower panels; Tables S1 and S2).

### Primary Metabolism

When larvae fed on JA-silenced plants, they regulated many genes involved in primary metabolism, which included transcripts that encode for proteins involved in the metabolism of carbohydrates, lipids, proteins and amino acids, nucleotides, energy, cofactors, vitamins, and glycans. *Ms* and *Hv* larvae that fed on JA-silenced plants regulated 3 and 1.4 times more transcripts for primary metabolism than larvae that fed on N- and N/PI-silenced plants, respectively. For example, *Ms* larvae that fed on JA-silenced plants regulated 2 times more transcripts for carbohydrate and amino acid metabolism, and 4 times more transcripts for carbohydrate metabolism than larvae that fed on N/PI- and N-silenced plants, respectively (Figure 3A, upper panel). The regulation was more pronounced in *Ms* larvae which regulated 1.9 times more transcripts than did *Hv* larvae in response to the overall JA-mediated defenses excluding N and N/PI (Figure 3A, inset, upper and lower panels). Although more transcripts for primary metabolism were regulated in *Ms* larvae, they regulated only specific pathways, while *Hv* larvae regulated transcripts for all primary metabolic pathways. For example, none of the transcripts for energy metabolism were regulated in *Ms* larvae that fed on defenseless plants (Figure 3A, upper and lower panels). Regulation in *Ms* larvae was highly diet-specific as observed by the regulation of transcripts for purine and pyrimidine metabolism, and cofactor and vitamin metabolism, which were not regulated by larvae that fed on N-silenced plants (Figure 3A, upper panel).

Regulation of genes within a given primary metabolic pathway in *Ms* larvae was also highly diet-specific. For example, transcripts for galactose metabolism were regulated only by *Ms* larvae that fed on N-silenced plants and transcripts for starch and sucrose, cysteine and tryptophan, and folate metabolism, were regulated only by *Ms* larvae that fed on JA-silenced plants (Table S1). However, all of these transcripts were regulated in *Hv* larvae that fed on N-, N/PI- and JA-silenced plants; in addition, they regulated a few transcripts not regulated by *Ms* larvae. For example, they regulated transcripts for energy metabolism, metabolism of other amino acids in addition to cysteine and tryptophan, and a few other pathways of carbohydrate and lipid metabolism not regulated by *Ms* larvae (Figure 3A, lower panel, Table S2). Overall, these results suggest that a suite of genes involved in primary metabolism are associated with the dramatic increase in larval growth that occurs when larvae feed on plants that lack all JA-mediated defenses.

### Secondary Metabolism

As expected, larvae that fed on plants silenced in JA-mediated defenses with altered levels of various secondary metabolites regulated some of the well-described detoxification enzymes, such as cytochrome P450, glutathione S-transferase and carboxylesterase, and other transcripts that code for proteins involved in the metabolism of plant secondary metabolites.

*Ms* larvae are known to be more tolerant to the synergistic defense of N and PI than *Hv* larvae [14], and this greater tolerance is reflected in the 1.2 fold greater number of transcripts regulated by *Hv* larvae than in *Ms* larvae when they fed on N/PI-silenced plants (Figure 3B, inset, upper and lower panels). However, *Ms* larvae regulated more of their transcripts for secondary metabolism in response to feeding on JA-silenced plants, and the response was much greater than was observed in *Hv* larvae. Hence, *Ms* larvae that fed on JA-silenced plants regulated 2–3 times more transcripts for secondary metabolism than did *Ms* larvae that fed on N- or N/PI-silenced plants (Figure 3B, inset, upper panel), and regulated 2 times more than *Hv* larvae that fed on JA-silenced plants (Figure 3B, inset, upper and lower panels). These results suggest that while *Ms* larvae have effectively adapted to dietary N and TPIs. *N. attenuata* produces other JA-regulated defenses, such as DTGs [49], which require substantial metabolic efforts on the part of the larvae to detoxify.

While the response varied considerably between *Ms* and *Hv* larvae, the two general patterns of regulation observed in genes involved in primary metabolism (up- and down-regulation, and diet-specific regulation) were also observed in the regulation of genes involved in secondary metabolism. First, while *Ms* larvae down-regulated genes in response to feeding on defenseless plants, *Hv* larvae up- and down-regulated genes from the same functional categories (Figure 3B, upper and lower panels). Second, while *Ms* larvae regulated transcripts in a diet-specific manner, *Hv* larvae regulated transcripts for similar functional categories when they fed on N-, N/PI- or JA-silenced plants. In addition, the diet-specific regulation observed in *Ms* larvae was also observed for transcripts of different pathways within secondary metabolism. For example, transcripts for the metabolism of terpenes (limonene and pinene) were regulated only by *Ms* larvae that fed on N-silenced plants, and transcripts for the metabolism of terpenoids and cytochrome P450s involved in the metabolism of xenobiotics were regulated only by *Ms* larvae that fed on JA-silenced plants (Table S1). In addition to these transcripts, *Hv* larvae also regulated transcripts for the metabolism of phenylpropanoid when they fed on N-, N/PI- or JA-silenced plants (Table S2).

### Peptidases, Hydrolases, and “Other Enzymes”

The patterns of regulation in peptidases, hydrolases and ‘other enzymes’, a category which includes the oxidoreductases, transferases and isomerases is represented in Figure 4A, B, and C.

*Ms* larvae that fed on JA-silenced plants regulated 2.6 times more transcripts for peptidases than did *Hv* larvae (Figure 4A, inset, upper and lower panels). *Ms* larvae that fed on JA-silenced plants also regulated 3–5 times more transcripts for peptidases than did larvae that fed on N/PI- and N-silenced plants, respectively (Figure 4A, inset, upper panel). For example, for serine peptidases, *Ms* larvae that fed on JA-silenced plants regulated 1.5–3 times more transcripts than larvae than that fed on N/PI- and N- silenced plants, respectively. This regulation of peptidases in *Ms* larvae was highly diet-specific. For example, they down-regulated a few metallo-peptidases and up-regulated a few omega-peptidases which were not regulated by *Ms* larvae that fed on N/PI- and N-silenced plants (Figure 4A, upper panel). The regulation of peptidases appeared to be a general response in *Hv* larvae; they regulated a similar number of transcripts with 72–80% peptidases commonly regulated across all diet types (Figure 4A, inset, lower panel). However, *Hv* larvae regulated 1.6 times more peptidase transcripts than did *Ms* larvae when both fed on N-silenced plants, but they regulated the same number of transcripts when they fed on N/PI-silenced plants (Figure 4A, inset, upper and lower panels). Interestingly, no transcripts for omega-peptidases were regulated by *Hv* larvae (Figure 4A, lower panel).

*Ms* larvae that fed on JA-silenced plants regulated 4–8 times more transcripts for hydrolases than did larvae that fed on N/PI- and N-silenced plants (Figure 4B, inset, upper panel); and the regulation was 6 times more than in *Hv* larvae that fed on JA-silenced plants (Figure 4B, upper and lower panels). *Ms* larvae also regulated 1.9 times more of these transcripts than did *Hv* larvae when they fed on N/PI-silenced plants (Figure 4B, inset, upper and lower panels). Unlike *Ms* larvae, *Hv* larvae that fed on defenseless plants regulated fewer transcripts for hydrolases. The generally observed pattern of up- and down-regulation was also observed for hydrolases (Figure 4B, upper and lower panels). This pattern of regulation was also true for transcripts categorized as ‘other enzymes’ but only for *Hv* larvae as *Ms* larvae did not differentially regulate this category of transcripts (Figure 4C, upper and lower panels). However, *Hv* larvae regulated similar number of theses transcripts when larvae fed on N-, N/PI- and JA-silenced plants (Figure 4C, inset, lower panel).

### Transporters

*Ms* larvae that fed on JA-silenced plants regulated 2.3–3.4 times more transcripts for transporters than larvae that fed on N- and N/PI-silenced plants (Figure 5, upper panel). However, *Hv* larvae regulated more transcripts for transporters when they fed on N-silenced plants (1.3 times more) than when they fed on N/PI- and JA-silenced plants (Figure 5, lower panel). Although both *Ms* and *Hv* larvae regulated the two ubiquitously occurring transporters, the ATP-binding cassette (ABC) superfamily and the major facilitator superfamily (MFS) proteins, involved in the transport of various substrates across extra- and intracellular membranes, the amount of regulation in *Hv* larvae was much less than in *Ms* larvae. *Ms* larvae regulated 16 times more MFS transporter transcripts than *Hv* larvae when they fed on JA-silenced plants, and 1.4–1.9 times more ABC transporter transcripts when they fed on N- and N/PI-silenced plants, respectively (Figure 5, upper and lower panel). Again, the general diet-specific pattern of gene regulation was also observed in the regulation of transporters. For example, while transcripts for all kinds of transporters were regulated by *Hv* larvae, transcripts for MFS transporters, and neurotransmitter and lipid transporters were regulated only in *Ms* larvae that fed on JA-silenced plants; transcripts for ABC transporters were regulated only by *Ms* larvae that fed on N- and NPI; and transcripts for ion-coupled transporters were regulated only by *Ms* larvae that fed on N/PI-silenced plants (Figure 5, upper and lower panels).

### Genetic Information Processing

This category included transcripts coding for regulatory proteins that take part in regulation at the DNA, RNA and protein level which facilitate the processing of genetic information. The diet-specific regulation of transcripts for genetic information processing was prominent in *Ms* larvae which differentially regulated transcripts for this functional category only when they fed on JA-silenced plants. For example, transcripts for proteins involved in protein targeting and degradation, transcription factors, proteins participating in the translation machinery and proteins that bind to other proteins were regulated only by *Ms* larvae that fed on JA-silenced plants (Figure 6, upper panel). In contrast to the patterns observed for the regulation of genes in other functional categories, *Ms* larvae up-regulated most of these transcripts. *Hv* larvae behaved as expected and both up- and down-regulated the transcripts in this category (Figure 6, lower panel). In *Hv* larvae, the response to N-silenced plants and JA-silenced plants were similar, but 1.4 times more than larvae that fed on N/PI-silenced plants (Figure 6, lower panel). *Ms* larvae regulated 1.4 times more transcripts for protein targeting and degradation when they fed on JA-silenced plants than did *Hv* larvae that fed on these plants (Figure 6, upper and lower panels).

We broadly classified transcripts for maintaining cellular communication, the immune system, the growth and development of cells and larvae, as cellular processes. While transcripts for cellular processes were regulated 3–6 times more by *Ms* larvae that fed on JA-silenced plants than larvae that fed on N- and N/PI-silenced plants, they were equally regulated by *Hv* larvae independent of their ‘silenced’ diet. However, *Hv* larvae regulated more transcripts than did *Ms* larvae and regulated transcripts not regulated by *Ms* larvae (Figure S1, upper and lower panels). For example, they regulated transcripts for cytoskeleton-related proteins, WD-repeat protein, ankyrin and troponin (Tables S1 and S2).

*Ms* larvae that fed on JA-silenced plants regulated more transcripts for environmental information processing (signal perception and transduction) than did larvae that fed on N- and N/PI-silenced plants and as expected, all transcripts were down-regulated (Figure S2, upper panel). Unlike *Ms* larvae, *Hv* larvae regulated similar number of transcripts for environmental information processing across the different diets (Figure S2, lower panel). *Hv* larvae, in addition to regulating transcripts regulated by *Ms* larvae (proteins part of the ErbB, calcium and mammalian target of rapamycin (mTOR) signaling pathways) also regulated transcripts encoding proteins that are part of the two-component, mitogen-activated protein kinase, Wnt and transforming growth factor beta (TGF-beta) signaling pathways (Tables S1 and S2).

## Discussion

Insect attack elicits defense responses in plants and these in turn elicit counter responses in the insects as they ingest the elicited plant material. To uncouple this cycle of response and counter response between plant and insect attackers, we fed larvae on isogenic plants that had been transformed to sequentially silence a suite of JA-mediated defenses (N, TPIs, and all JA-mediated defenses) and analyzed the transcriptional responses in neonates with an unbiased experimental protocol. We selected two insect species that commonly attack *N. attenuata* in nature [14], [51]: the oligophagous *Ms* larvae and the polyphagous *Hv* larvae, as examples of specialist and generalist lepidopteran herbivores, respectively. *Ms* adults preferentially oviposit on solanaceous plants, but have recently been reported to feed on 2 *Proboscidea* species, a non-solanaceous plant [62], while adult *Hv* females oviposit on plant species belonging to more than 14 taxonomically diverse families, including solanaceous taxa (tobacco, tomato etc.) [63]. Although *N. attenuata* plants elicit slightly different transcriptional signatures in response to attack from these two species, there is considerable overlap in the defense genes activated [51] and by restraining the host plant's defense response by transformation, we uncoupled the response cycle between plant and insect.

While our choice of “specialist” and “generalist” taxa for the comparison is clearly confounded by their different phylogenetic histories, the growth performances of these two species on *N. attenuata* have been studied and are consistent with the expectations of greater tolerance of the specialist (*Ms*) to the specific defense metabolites of *N. attenuata* than the generalist (*Hv*) [51]. Similar results have been reported from other, equally phylogenetically uncontrolled comparisons. For example, *Ms* larvae increased their mass by 1.5-fold when they fed on N-silenced plants in comparison to WT plants while another generalist larvae (*Spodoptera exigua*) increased their mass by 4-fold compared to WT-fed larvae [50]. The objective of the analysis was to determine if an herbivore that has evolved tolerance against the defenses of its host plant would regulate fewer genes than a non-adapted species would or more specifically if *Ms* would regulate a few, but specific genes and *Hv* would regulate more of its genes in response to the diverse array of *N. attenuata's* defenses. The results were consistent with this expectation. Since both generalists and specialists had been previously shown to perform better on JA-silenced plants [46], [64], we expected that the greater the number of defenses silenced in the host plant (JA>N/PI>N), the greater the number of transcripts that would be regulated in both herbivores, but the specificity of the regulation would differ, so that the specialist would display a more diet-specific response in comparison to the generalist. Again the results of the study were consistent with these expectations.

**Figure 7:**
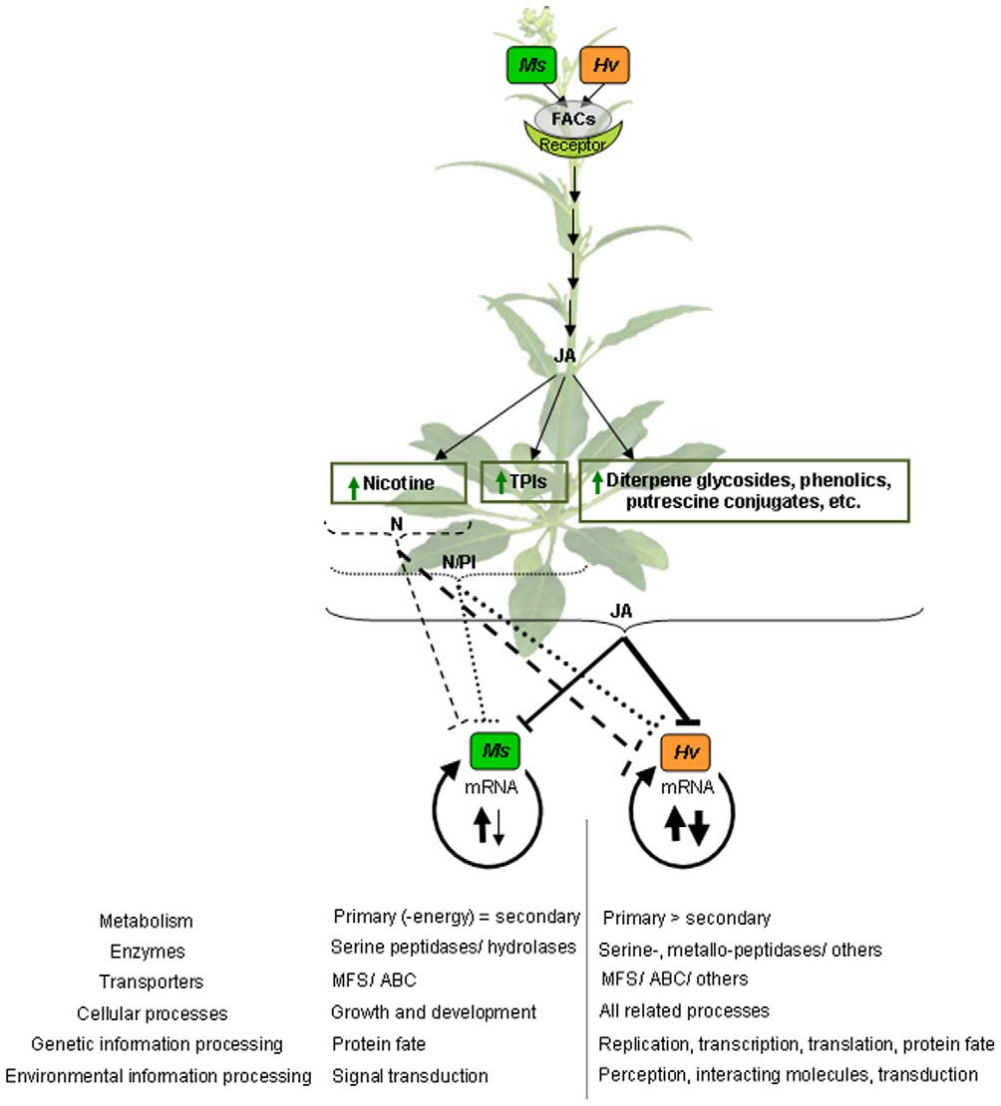
Summary of the pattern of transcriptional regulation in a specialist (*Ms*) and generalist (*Hv*) herbivore as they feed on *N. attenuata* host-plants deficient in JA-mediated defenses, defenses that are normally activated in response to herbivore attack. Feeding by *Ms* and *Hv* larvae elicits JA-mediated defense responses. In response, the specialist herbivore (*Ms*) fine-tunes its transcriptome by activating a few but specific transcripts; however, the generalist herbivore (*Hv*) regulates a large portion of its transcriptome. Both species responded similarly to JA-mediated defenses with more genes regulated in larvae feeding on JA-silenced plants followed by N/PI- and N-silenced plants, but the effect of these defenses elicits a larger transcriptional response in *Hv* larvae than in *Ms* larvae. The thickness of the lines and arrows reflects the extent of transcriptional changes elicited in herbivores in response to the ingestion of plant material containing anti-herbivore defenses.

While our general expectations for the number of transcripts regulated between *Hv* and *Ms* larvae feeding on progressively defenseless plants were met, there were considerable differences (Figure 7) which can be distilled to two additional general patterns: *Ms* larvae largely down-regulated its genes while *Hv* larvae both up- and down-regulated its genes from the same functional categories, and *Ms* larvae regulated its genes in a diet-specific manner while *Hv* larvae regulated similar transcripts across all diet types. These two patterns are also consistent with general expectations for differences between generalists and specialists. The adaptations of specialists likely entail the regulation of specific genes to counter their host plant's responses and since our hybridizations compared the responses of larvae feeding on defenseless plants to larvae feeding on WT plants, the pattern of gene regulation we observed (down-regulation) is consistent with an up-regulation of detoxification responses associated with the ingestion of larger quantities of feeding-elicited defenses. Similarly, generalist herbivore species would be expected to counter the host plant's defense responses with a more generalized counter response. The details of the specific genes regulated provide insights into the mechanisms by which the neonates adjust their physiologies to diets deficient in the N, N/PI and JA-elicited responses in *N. attenuata*.

Numerous mechanisms have been suggested as being responsible for the remarkable tolerance of *Ms* larvae to dietary N intake. Three remain viable hypotheses: rapid detoxification by P450 enzymes [59], [60], rapid excretion [58], [65], [66], [67], and intrinsic insensitivity of *Manduca's* central nervous system to N [68]. A fourth mechanism, modifications in N's binding to *Ms* nicotinic acetylcholine receptors, has recently been ruled out [66]. Although all the three mechanisms have found experimental support, their relative importance remains unclear. The N-inducible expression of a variety of P450s is also observed in *Hv* larvae [69]. Although less is known about the mechanisms that operate in *Hv* larvae, an inducible detoxification and excretion system seem to operate in *Ms* larvae that helps them tolerate N. However, it is not known if these mechanisms also operate in neonates, as most studies used late instar larvae. Our transcriptional analysis suggests that alkaloid excretion, as indicated by the exclusive down-regulation of members of the ABC superfamily of transporters in *Ms* larvae that fed on N/PI- and N-silenced plants, rather than N detoxification by P450 enzymes (which were not regulated), may be more important for the tolerance of *Ms* neonates to N. *Hv* neonates were found to regulate (both up- and down-regulate) a few transcripts for ABC transporters and P450s, but *Ms* larvae regulated 1.4–1.9 times more transcripts for ABC transporter than did *Hv* larvae when they fed on N- and N/PI-silenced plants, respectively.

N functions as an antifeedant, causing N-adapted herbivores to curtail consumption likely due to the costs of N detoxification [33], [70], [71]. PIs decrease insect growth by inhibiting gut proteases and potentially reducing the availability of essential amino acids [72]. Adaptation to the ingestion of dietary PIs has been documented in a number of insects [73], [74], [75] and involves one or a combination of the following strategies: the overproduction of existing inhibitor-sensitive digestive proteases [76], [77], [78], [79]; increased expression of inhibitor-insensitive protease isoforms [74], [77], [80], [81], [82], [83]; activation of proteases that hydrolyze plant PIs [76], [83], [84]; and compensatory feeding [74], [85]. While specialists are likely to have evolved more specific means of coping with diets rich in PIs, generalists are more likely to have evolved more generalized behavioral responses to impaired digestive function [86], [87]. This is consistent with our observation that *Hv* larvae regulated 72–80% of its peptidases (belonging to different classes) as a general response to feeding on plants silenced in N, N/PI and JA, while *Ms* larvae largely down-regulated serine peptidases when they fed on plants silenced in PIs (N/PI- and JA-silenced plants). We also observed, that *Ms* larvae fed on N/PI-silenced plants down-regulated a larger number of serine peptidases than did larvae that fed on N-silenced plants, a result consistent with the expectation that N complements the defensive function of TPIs in *N. attenuata* by restraining the compensatory feeding of herbivores [14]. Although it is difficult to conclude without the results of transcriptional changes in larvae that fed on plants silenced only for TPIs, we infer that *Ms* and *Hv* neonates employ similar strategies when they ingest host plant TPIs.

JA elicitation increases the production of some direct defenses, such as N and TPIs, against which *Ms* larvae have evolved tolerance. However, *Ms* larvae are still susceptible to other JA-mediated defenses, such as diterpene glycosides (DTGs) which accumulate to concentrations equivalent to that of starch in herbivore attacked tissues. *Ms* larvae that fed on plants silenced in DTG production gained 3 times more mass than larvae that fed on N- or PI-silenced plants [49], [88].

Herbivore attack to *N. attenuata* elicits a massive reprogramming of their transcriptome, proteome and metabolome that reflects the re-allocation of resources from primary metabolism to fuel secondary metabolism [89]. This metabolic readjustment can reduce the nutritional value of the leaf consumed by herbivores, and may function as an alternative defensive response. As JA elicitation is known to reduce the shoot quality by reducing total sugar and amino acids [90], photosynthetic proteins [91], and increasing the activity of enzymes that degrade essential amino acids in herbivores [92]. *Ms* larvae that fed on JA-silenced plants down-regulated MFS transporters that are thought to function in transport of sugar [93], [94] and toxic compounds [95]. A part of the rapid growth of both generalists and specialists on JA-silenced plants [46], [64] may reflect these changes in primary metabolism. The dramatic up-regulation of primary metabolism genes in both *Ms* and *Hv* larvae that fed on JA-silenced plants may reflect a similar shift in metabolic priorities from defense-detoxification to growth-related processes and may reflect the degree to which the ingestion of host plant defenses constrains the growth potential of larvae by the metabolic costs of their detoxification. This interpretation is consistent with the observation that *Ms* larvae that fed on JA-silenced plants down-regulated genes involved in secondary metabolism (cytochrome P450s, GSTs and COEs) while up-regulating genes involved in carbohydrate, lipid, nucleotide, cofactor and vitamin metabolism. However, the possibility that *Ms* and *Hv* larvae may eavesdrop on its host plant's JA signaling to regulate its detoxification enzymes represents an alternative explanation for the large transcriptional regulation observed in larvae that fed on JA-silenced plants. Cytochrome P450s are known to play a role in the detoxification of plant chemicals in *Ms* larvae [96] and generalist *Helicoverpa species*, in which they are elicited by the ingestion of host plant signal molecules, JA and SA [97].

The inferences reported in this study are drawn from a single pair of generalist and specialist herbivores and will need to be verified in additional comparisons, preferably ones sharing a more similar evolutionary history. However, the inferences provided by this study provide many hypotheses, which can be rigorously tested once efficient plant-mediated RNAi systems have been developed to silence the expression of the diet-induced changes in transcripts in the insects [98]. In this way, the plant-insect transcriptional duet can be uncoupled on the insect side of the equation, and would nicely complement to the uncoupling conducted in this study on the plant side of the equation. Silencing of the candidate genes that were found to be strongly regulated in this study such as MFS and ABC transporters, some of the cytochrome P450s, in combination with metabolomic studies [99] will likely provide insights into the mechanism by which neonates rapidly adapt to the ingestion of host-derived defenses, or anticipate the ingestion of these toxins.

## Materials and Methods

### Plant Material and Growth Conditions

We used isogenic lines of *Nicotiana attenuata* (Solanaceae), obtained after 22 generations of inbreeding a genotype collected from a burn in southwestern Utah, for transformations and untransformed wild type (WT) plants. We transformed WT plants with RNAi and antisense constructs to silence nicotine (N) production, N production together with trypsin protease inhibitor (TPI) and jasmonate (JA) biosynthesis. All transformants were homozygous for a single insertion and have been fully characterized in previous publications. Briefly, for plants silenced in JA biosynthesis, we used a fragment of NaLOX3, a key enzyme of JA biosynthesis, expressed in an antisense (as) orientation in the WT genotype, as characterized in [46]. These *as-lox3* plants (line A300) accumulate only 35% and 50% of the JA that WT plants do after mechanical wounding and treatment with water, and mechanical wounding and the application of OS, respectively. By silencing JA signaling, the plants are impaired in all JA-mediated defenses, which include N, TPIs, diterpene glycosides (DTGs), and herbivore-induced volatile organic compounds (VOCs) that function as indirect defenses [46]. In plants silenced for N, line A03-108, in which a consensus fragment of *N. attenuata's* two *putrescine N-methyl transferase* (*pmt*) genes, which are regulatory enzymes of nicotine biosynthesis, was expressed in an inverted-repeat (ir) orientation, as characterized in [50]. The *ir-pmt* plants have drastically reduced transcripts of both PMT genes and produce no detectable quantities of N. To produce plants silenced for both N and TPIs (N/PI), homozygous T2 generation *ir-pmt* plants were re-transformed with ir constructs of NaTPI, as characterized in [14]. The *ir-pmt/pi* plants (line A04-103) are completely silenced in N and TPI production. All transgenic lines were morphologically indistinguishable from WT plants. Seeds were germinated in diluted liquid smoke solution as described in [100], and seedlings were transplanted into soil-containing pots in a glasshouse under the conditions described in [101] with 1000–1300 µmol m^−2^s^−1^ photosynthetic photon flux density supplied by 450 W Na-vapor high-intensity discharge bulbs.

### Herbivores of *N. attenuata*

Eggs of *Manduca sexta* (*Ms*) and *Heliothis virescens* (*Hv*) from in-house reared populations were kept in a growth chamber (Snijders Scientific, http://www.snijders-tilburg.nl) at 26°C 16 h light, 24°C 8 h darkness, until the larvae hatched. Freshly hatched 20–25 *Ms* and 35–40 *Hv* neonates were placed on fully developed leaves of single rosette-stage *N. attenuata* plants for each genotype. After 24 h of feeding on each genotype, 15 *Ms* larvae and 30 *Hv* larvae were collected in 1.5 ml Eppendorf micro-centrifuge tubes, flash-frozen in liquid nitrogen and stored at −80°C until RNA was extracted using the TRIzol^®^ reagent from Invitrogen (Carlsbad, CA, USA). This whole larvae RNA was used in the hybridizations of the cDNA microarrays.

For the creation of the cDNA microarrays, midgut tissues were isolated from neonates of both species that had fed for 24 h on WT plants elicited with 250 µg methyljasmonate (MeJA) in 20 µl of lanolin, three days prior to larval feeding. Three WT plants elicited with MeJA were maintained for each larval species with 20 *Ms* and 40 *Hv* neonates feeding on each plant. Approximately 40 *Ms* larvae and 100 *Hv* larvae were harvested after 24 h of feeding and dissected in ice-cold 1x PBS buffer (Applied Biosystems/Ambion, TX, USA) under a stereomicroscope (Carl Zeiss, Jena, Germany) by first de-heading the caterpillars (up to the third pair of forelegs) and excising the abdomen with a sterile scalpel. As a result of the incisions made at the anterior and posterior ends, the midguts were expelled from the center portion of the larval carcass. The dissected midguts were gently pressed to remove gut contents and free the guts from the adhesive muscle/fat tissue. Immediately following the dissections, individually isolated tissues were pooled, collected in 1.5 ml Eppendorf micro-centrifuge tubes, flash-frozen in liquid nitrogen and stored at -80°C until RNA was extracted. Total RNA was isolated from whole larvae and midgut tissues using the TRIzol^®^ reagent from Invitrogen and used for cDNA library construction.

### cDNA Library Construction

A normalized midgut cDNA library was constructed using total RNA isolated from *Ms* and *Hv* larvae that had fed on pre-elicited WT *N. attenuata* plants as described above. Double-stranded cDNA was synthesized using the Creator^™^ SMART^™^ cDNA library construction kit from Clontech (Mountain View, CA, USA). Following double-stranded cDNA synthesis, normalization was performed with a duplex-specific nuclease (DSN) treatment included in the Trimmer-Direct cDNA normalization kit from Evrogen (Moscow, Russia). The manufacturer's protocol was followed for both procedures except for one alteration using the cDNA library construction kit. Instead of cloning the (PCR-amplified) fragments into the provided phage vector pDNR-LIB (GenBank DQ666274), they were directionally cloned into the vector pUCLIB (2.9 kb), which was obtained by fusing the polylinker of pDNR-LIB as 0.2 kb *Eco*RI-*Hin*dIII-fragment to the 2.6 kb *Eco*RI-*Hin*dIII-fragment of pUC19 (GenBank M77789). Colony picking, plasmid DNA isolation and subsequent PCR-amplification for 5000 randomly selected clones were performed at Qiagen GmbH (Hilden, Germany).

### cDNA-Array Fabrication, Hybridization, and Quantification

A total of 5000 randomly selected and PCR-amplified clones from each of the normalized *Ms* and *Hv* cDNA libraries were spotted on epoxy coated slides as described in [102]. For each cDNA, two PCR fragments, each with a 5′ -aminolink on either strand, were synthesized, and each PCR fragment was spotted four times. Hence, each gene was represented by two independent PCR fragments, which, in turn, were spotted in quadruplicate. The hybridizations were performed as described by [102] with the following modifications: 10 µg of total RNA isolated from larvae that had fed on WT and transgenic lines were reverse-transcribed without mRNA isolation. The first step of the pre-hybridization washing was performed with 2% (w/v) SDS instead of a Triton X-solution. Microarrays were scanned with an array scanner (GMS 418, MWG, Ebersberg, Germany) and spot intensities (SIs) for Cy3 and Cy5 were extracted from image files using the AIDA software, version 4.03 (Raytest, http://www.raytest.com/). Normalization and statistical analysis of each microarray were performed as described in [103]. For a simplified visualization, down-regulated transcripts with a ratio smaller than 1 were transformed by dividing ‘−1’ by the Cy3/Cy5-ratio.

### EST Sequencing, Processing, and Functional Annotation

Clones that were differentially regulated in *Ms* and *Hv* larvae in response to feeding on defenseless plants, as identified by microarray studies, were selected and subjected to single-pass sequencing using a 5′ vector primer. DNA sequencing was performed by Qiagen GmbH (Hilden, Germany). Sequence information was stored in chromatograph trace files, and Phred was used to perform base-calling [104]. Flanking vector and adaptor sequences were trimmed using Cross-match (http://www.sanger.ac.uk/Software/) and Lucy [105], while low-quality bases (quality score <20) were cleaned at both sequence ends using our custom program. RepeatMasker (http://ftp.genome.washington.edu/) was used to mask repeated sequences, and the masked sequences were further screened to remove contaminating sequences from bacteria and viruses using BLASTN [106]. The cleaned expressed sequence tags (EST) were then subjected to clustering using the TIGR software TGI Clustering tool (TGICL) [107]. The clustering was performed by a modified version of NCBI's megablast. EST sequences were assigned to clusters based on identity: the clustering parameters were 98% minimum percent identity for overlaps, for a minimum overlap length of 40 nt and a maximum length of unmatched overhangs of 20 nt. The cluster names corresponded to the name of the first EST sequence assigned to the cluster. Sequences from each cluster were assembled into consensus sequences (contigs) using the CAP3 assembly program available in TGICL. High quality assembled ESTs were annotated using BLASTX through NCBI and our local BLAST server with a cut-off E-value of 1e-10. The sequences reported in this study have been deposited in GenBank under accession numbers FK816474-FK817136 (*Ms*) and GT054264-GT056056 (*Hv*). The sequences were mapped to KEGG (Kyoto Encyclopedia of Genes and Genomes) biochemical pathways according to the EC distribution in the pathway database [108] and assigned the KO IDs (KEGG orthology); sequences which did not match any KO IDs were manually searched against the Uniprot and functionally annotated. While the microarray data presented here are fully MIAME compliant, the raw data has not been deposited in a MIAME compliant database because not all spotted clones on the arrays have been sequenced (only the significantly regulated clones were sequenced). The MIAME-compliant publically available databases require that all clones spotted on cDNA arrays are sequenced. Hence we present all of the raw data, list of clone IDs with expression ratios and functional annotation, in Excel files as supplementary material (Table S1 (*Ms*) and S2 (*Hv*)).

## Supporting Information

Figure S1 Fewer genes for cellular processes were regulated in *Manduca sexta* than in *Heliothis virescens* larvae that fed on plants silenced for jasmonate (JA) signaling, N/PI, and N defenses. The highest degree of regulation for these genes was found in *M. sexta* larvae that fed on JA-silenced plants and *H. virescens* larvae that fed on N-silenced plants. Open bars: up-regulated (ER >1.5); solid bars: down-regulated (ER <−1.5) by *M. sexta* (upper panel) and *H. virescens* (lower panel).
(0.04 MB TIF)

Figure S2 All the genes coding for environmental information processing were down-regulated in *Manduca sexta* larvae, while in *Heliothis virescens* larvae they were both up- and down-regulated. Both species regulated a high percentage of genes when they fed on jasmonate-silenced plants. Open bars: up-regulated (ER >1.5); solid bars: down-regulated (ER <−1.5) by *M. sexta* (upper panel) and *H. virescens* (lower panel).
(0.03 MB TIF)

Table S1 Genes transcriptionally regulated by specialist *Manduca sexta* larvae in response to jasmonate-mediated defense response of *Nicotiana attenuata*.
(0.09 MB XLS)

Table S2 Genes transcriptionally regulated by generalist *Heliothis virescens* larvae in response to jasmonate-mediated defense response of *Nicotiana attenuata*.
(0.83 MB XLS)
