# Supplementary figures and images for: Unbiased Transcriptional Comparisons of Generalist and Specialist Herbivores Feeding on Progressively Defenseless Nicotiana attenuata Plants
Source: PLoS One. 2010 Jan 15;5(1):e8735. doi: 10.1371/journal.pone.0008735 (PMC2806910; doi:10.1371/journal.pone.0008735)

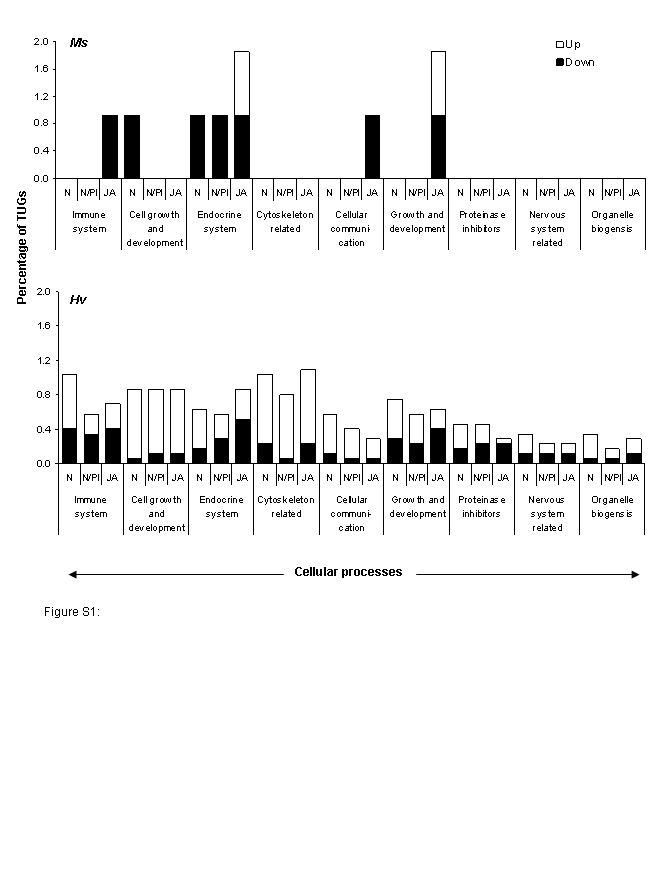

Supplement: Figure S1 — Fewer genes for cellular processes were regulated in Manduca sexta than in Heliothis virescens larvae that fed on plants silenced for jasmonate (JA) signaling, N/PI, and N defenses. The highest degree of regulation for these genes was found in M. sexta larvae that fed on JA-silenced plants and H. virescens larvae that fed on N-silenced plants. Open bars: up-regulated (ER >1.5); solid bars: down-regulated (ER <−1.5) by M. sexta (upper panel) and H. virescens (lower panel). (0.04 MB TIF) [file pone.0008735.s001.tif]

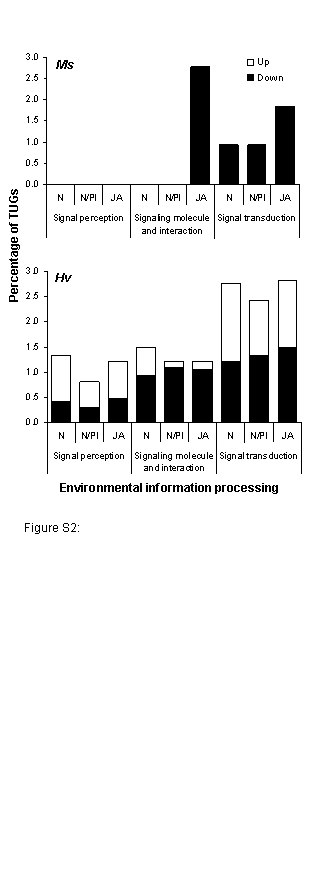

Supplement: Figure S2 — All the genes coding for environmental information processing were down-regulated in Manduca sexta larvae, while in Heliothis virescens larvae they were both up- and down-regulated. Both species regulated a high percentage of genes when they fed on jasmonate-silenced plants. Open bars: up-regulated (ER >1.5); solid bars: down-regulated (ER <−1.5) by M. sexta (upper panel) and H. virescens (lower panel). (0.03 MB TIF) [file pone.0008735.s002.tif]
